# Parvalbumin-Neurons of the Ventrolateral Hypothalamic Parvafox Nucleus Receive a Glycinergic Input: A Gene-Microarray Study

**DOI:** 10.3389/fnmol.2017.00008

**Published:** 2017-01-23

**Authors:** Viktoria Szabolcsi, Gioele W. Albisetti, Marco R. Celio

**Affiliations:** Anatomy and Program in Neuroscience, Department of Medicine, University of FribourgFribourg, Switzerland

**Keywords:** parvalbumin, laser capture microdissection, glycine receptor α2, Allen Brain Atlas Database of *in-situ* hybridizations (ABA-ISH), BrainStars (B^*^) microarrays, PV1-Foxb1, parvafox, GlyT2-EGFP

## Abstract

The ventrolateral hypothalamic parvafox (formerly called PV1-Foxb1) nucleus is an anatomical entity of recent discovery and unknown function. With a view to gaining an insight into its putative functional role(s), we conducted a gene-microarray analysis and, armed with the forthcoming data, controlled the results with the Allen databases and the murine BrainStars (B^*^) database. The parvafox nucleus was specifically sampled by laser-capture microdissection and the transcriptome was subjected to a microarray analysis on Affymetrix chips. Eighty-two relevant genes were found to be potentially more expressed in this brain region than in either the cerebral cortex or the hippocampus. When the expression patterns of these genes were counterchecked in the Allen-Database of *in-situ* hybridizations and in the B^*^-microarray database, their localization in the parvafox region was confirmed for thirteen. For nine novel genes, which are particularly interesting because of their possible involvement in neuromodulation, the expression was verified by quantitative real time-PCR. Of particular functional importance may be the occurrence of glycine receptors, the presence of which indicates that the activity of the parvafox nucleus is under ascending inhibitory control.

## Introduction

The parvafox nucleus (formerly known as PV1-Foxb1 nucleus) has been identified and described as a bilateral chord of parvalbumin (Parv)-positive neurons in the murine ventrolateral tuberal hypothalamus (Celio, 1990; Mészár et al., 2012; Bilella et al., 2014). It is sandwiched in the horizontal plane between the optic tract and the fornix, and in mice it extends for a total length of about 1 mm. Parv-positive cells of the parvafox nucleus are immunoreactive for glutamate (Mészár et al., 2012) and express the gene *Slc17a6* encoding the vesicular glutamate transporter 2 (Vglut2) (Girard et al., 2011). Hence, unlike the usual GABA-phenotype (Celio, 1986), the Parv-positive neurons of the parvafox nucleus utilize the excitatory neurotransmitter glutamate.

The efferent connections of the Parv-positive sub-population of neurons in the parvafox nucleus are almost exclusively directed toward a small cylindrical zone lying ventral to the aqueduct in the periaqueductal gray matter (PAG) (Celio et al., 2013). The targets of the Foxb1-cell subpopulation of the parvafox nucleus are likewise located in and around the PAG, but occur also in a few other cerebral sites (Bilella et al., 2016). The inputs to the parvafox nucleus, as studied by trans-synaptic retrograde transport of rabies-virus, derive from a wide variety of brain sites, including orbitofrontal cortex, piriform cortex, olfactory tubercle, nucleus accumbens, caudoputamen, globus pallidus, diagonal band nucleus (NDB), substantia innominata (SI), bed nuclei of the stria terminalis (BST), anterior amygdalar area, central and medial amygdalar nucleus, thalamus [(lateral dorsal nucleus of the thalamus (LD), mediodorsal nucleus of the thalamus (MD)], midbrain (PAG, ventral tegmental area, midbrain reticular nucleus), pons, medulla oblongata [Gigantocellular reticular nucleus (GRN), Intermediate reticular nucleus (IRN)] (manuscript in preparation). The functions of this lateral hypothalamic parvafox nucleus are as yet unknown.

The identification of genes that are enriched in the neurons of the parvafox nucleus could reveal the presence of components that might provide an insight into the role(s) of this brain entity. A genome-wide molecular characterization of the parvafox nucleus has been already undertaken by a screening of the Allen Brain Atlas Database of *in-situ* hybridizations (ABA-ISH) (Girard et al., 2011). This former study disclosed 76 genes as being potentially co-expressed with Parv in the neurons of the parvafox nucleus, a large number of them being common to all Parv-positive neurons in the brain (e.g., those that are implicated in the regulation of potassium–channels). Somewhat surprisingly, only a few of the genes that encode neuromodulators or their receptors were found to be expressed in the parvafox nucleus.

With a view to complementing these early data and to disclosing genes encoding neuropeptides, neurotransmitters and their receptors, the entire transcriptome of the parvafox nucleus was subjected to a gene-microarray of laser-capture microdissected murine tissue. Armed with these data, we scrutinized the Allen Database of *in-situ* hybridizations (ABA-ISH; http://www.brain-map.org/) (Lein et al., 2007) and the BrainStars (B^*^)-Database of gene arrays (Kasukawa et al., 2011; http://brainstars.org/). The most significant outcome of this gene-array investigation was the discovery of glycine-receptor alpha subunits expressed in the parvafox nucleus.

## Materials and methods

### Mice

Adult C57BL/6 and GlyT2-EGFP mice of both sexes were used in this study. GlyT2-EGFP mice were a kind gift of Prof. Hanns Ulrich Zeilhofer (University of Zürich, Neuropharmacology). All animal experiments were carried out in accordance with the institutional guidelines of the University of Fribourg and with the permission of the Swiss federal and cantonal committee on animal experimentation.

### Gene-microarray analysis

Six C57BL/6 mice were killed by cervical dislocation. The brains were rapidly removed, mounted on cork platforms in OCT (Sakura Tissue-Tek), and frozen on pulverized dry ice. Prior to sectioning in a cryostat, the samples were stored at −80°C. Starting from the ventral surface, the brains were cut into 16-μm-thick horizontal sections at −18°C until the fornix was reached. At this point, the sections were respectively mounted in an alternating manner on MMI-membranes (Molecular Machines & Industries, Glattbrugg, Switzerland) and Superfrost-Plus glass slides (Menzel-Gläser, Braunschweig, Germany). The MMI-mounted sections were chemically fixed for 5 min in 100% ethanol at −18°C, air dried for 1 h in the cryostat chamber, likewise at −18°C and stored at −80°C for various periods of time until the time of laser-beam-aided microdissection. The Superfrost-Plus-mounted sections were chemically fixed for 10 min at ambient temperature in 4% paraformaldehyde diluted in phosphate-buffered saline (0.1M, pH 7.3). These sections were incubated overnight at 4°C with the horseradish-peroxidase-conjugated lectin *Vicia villosa* (EY Laboratories, San Mateo, California, USA) at a concentration of 20 μg/ml in 0.1 M Tris-buffered saline containing 0.1 mM MnCl_2_, 0.1 mM CaCl_2_, 0.1 mM MgCl_2_ and 0.1% Triton-X. The presence of the lectin staining was revealed after treatment with diaminobenzidine / hydrogen peroxide. Lectins such as *Vicia villosa* are markers of the perineuronal nets (Celio and Blümcke, 1994), which are abundant around the Parv-immunopositive neurons of the rodent parvafox nucleus (Mészár et al., 2012). The pattern of lectin staining on the Superfrost-Plus-mounted sections aided the localization of the parvafox nucleus on the adjacent, MMI-membrane-mounted ones.

Using vessels as landmarks, the corresponding parvafox region on the MMI-membranes was excised using the CellCut-Plus laser microdissection apparatus (MMI, Glattbrugg, Switzerland) of the Cellular Imaging Facility (CIF) at the University of Lausanne. The previously drawn trajectory was automatically excised using the precisely focused UV-laser beam. The dissected tissue samples were collected in special tubes bearing an adhesive surface on the interior of the cap (MMI, Glattbrugg, Switzerland). Finally, 350 μl of lysis buffer [Buffer RLT (Qiagen), supplemented with 1:100 β-mercaptoethanol] was added to each tube and the samples were immediately frozen at −80°C. The same protocol was used to extract tissue from regions encompassing the cerebral cortex (piriform and entorhinal cortex) and the hippocampus (CA2–CA3 area). These regions were arbitrarily chosen as a basis for comparison in so far they contain GABAergic, Parv-positive interneurons. Hence, from each murine brain, three regions were excised: the parvafox nucleus, the cortex and the hippocampus.

The entire RNA-pool of the microdissected cryosections was purified using the RNAeasy Plus Micro Kit (Qiagen, Hilden, Germany). The quantity and the quality of the RNA-extracts were respectively monitored in a NanoDrop 2000 spectrophotometer (Thermo Scientific, Wilmington, DE, USA) and an Agilent 2100 bioanalyzer (Agilent Technologies, Palo Alto, California, USA). For the final gene-array we used the tissue of two mice (m355 and m511) from which the RNA-extract was best preserved and at the highest concentrations. The samples of these animals were analyzed separately so as to furnish independent replicates. The RNA-samples were stored at −80°C until the time of amplification using the NuGEN Ovation® Pico WTA System V2 (NuGEN Technologies, San Carlos, California, USA) as previously described (Szabolcsi and Celio, 2015). The whole-transcript amplification, the labeling process and the gene-chip hybridization on an Affymetrix Mouse Gene 2.0 ST Array (Affymetrix, Santa Clara, California, USA) were performed in the Genomic Technologies Facility (GTF) at the University of Lausanne (UNIL). Excel spreadsheets containing the extracted data were produced alongside the original files that were generated using the Affymetrix GeneChip® System.

### Quantitative real-time-PCR

Verification of the results of the gene microarray was performed by quantitative real-time-PCR (qRT-PCR). The same cDNA samples as used for the microarray analyses were subjected to SYBR qRT-PCR. Nine particularly interesting genes found enriched in the parvafox nucleus of the mice in our microarray study (*Cbln1, Drd2, Ephb1, Foxb1, Glra1, Glra2, Glra3, Npb, Npsr1*) were selected for the assay. Actin beta (*Actb*) was chosen as internal normalization gene. The qRT-PCR reactions were performed using Rotor-Gene Strip Tubes (Starlab Switzerland, Muri, Switzerland) in FastStart Essential DNA Green Master (Roche, Rotkreuz, Switzerland) containing 0.5 μM gene-specific primers and 5 ng cDNA/tube. The sequence of the primer pairs is listed in Supplementary Table 3. Assays were performed with a Rotor-Gene 6000 by Corbett Research (QIAGEN, Basel, Switzerland). The cycling program initiated with a 10 min hold at 95°C, and the cycles consisted of 20 s hold at 95°C, 20 s hold at 60°C, 20 s hold at 72°C, with 40 repeats. All samples were run in triplicates and C_T_ values were averaged for final quantification.

### Immunohistochemistry

Three C57BL/6 and two GlyT2-EGFP mouse brains were fixed by perfusion through the heart of 0.9% NaCl solution followed by cold 4% paraformaldehyde (PFA) diluted in phosphate-buffered saline (0.1 M, pH 7.3) and post-fixed overnight in the same buffer. The brains were transferred to cryoprotectant solution (30% saccharose in Tris-buffered saline 0.1 M, pH 7.3) and cut into 40 μm-thick coronal sections with a freezing microtome (Frigomobil, Reichert-Jung, Vienna, Austria). C57BL/6 sections were incubated for various length of time (1 to 3 days) with Glyrα2 antibody (Santa Cruz; Dallas, TX, USA; sc 20133) diluted 1:250 to 1:1000 (together with the monoclonal Parv antibody 235 (1: 1000; Swant Inc., Marly, Switzerland). The specificity of the Glyrα2 antibody was tested on 40 μm-thick sections of the prefrontal cortex derived from adult Glra2-KO mice, a kind gift of Prof. Bert Brône's lab (Morelli et al., 2016) (data not shown). GlyT2-EGFP sections were incubated overnight with monoclonal anti-GFP (1:1000, Molecular Probes, Thermo Fisher Scientific, Reinach, Switzerland) and polyclonal Parv antibody (PV-25, 1:1000, Swant Inc., Marly, Switzerland). Glyrα2-immunolabeling was revealed with the fluorescent avidin-biotin method (Vector labs), whereas GFP- and Parv-immunolabeling was detected with fluorophore conjugated secondary antibodies (Jackson Immunochemicals). Images were taken by a Leica epifluorescence microscope (DM6000B) and a Leica TCS SP5 confocal laser microscope. Image analyses and three-dimensional reconstructions were performed with the Imaris 8.3 software module (Bitplane, Zürich, Switzerland).

### Immunoblot

To obtain hypothalamic protein extracts, two C57BL/6 mice were subjected to cervical dislocation, followed by removal of the brains and manual excision of the hypothalamic, cortical and hippocampal regions. Total protein content of the excised tissue was extracted by ultrasonication in Tris-EDTA solution. Protein extracts were subjected to 10% SDS-gel-electrophoresis (50 μg protein) followed by transfer on nitrocellulose paper and immunoblot using an antibody directed against Glyrα2 (Santa Cruz, sc20133) diluted 1:500. The bound antibody was revealed with an HRP-labeled secondary antibody and the reaction amplified by chemiluminescence.

### Informatics

Data were pre-processed and normalized using the robust multi-array average (RMA) approach and the software “Expression Console” provided by Affymetrix (version 1.2.1.20). The data were evaluated using the programming language R (version 2.14.2) and Partek® Genomics Suite™ software (Partek Inc., St. Louis, Missouri, USA). Gene lists were prepared by the Genomic Technologies Facility of Lausanne using the Bioconductor package limma in R. NCBI Gene Expression Omnibus (GEO) database accession number of the gene microarray data is GSE92955.

The data gleaned from our gene-microarray analysis were compared to those derived by mining the B^*^-database and the Allenminer screening (v1.5) (Davis and Eddy, 2009), in order to retrieve the three-dimensional expression files that were prepared from the ABA-ISH database (Lein et al., 2007) in a former study (Girard et al., 2011).

### Mining of the BrainStars (B^*^) database

BrainStars (B^*^) (http://brainstars.org/) is a quantitative expression database of the adult murine brain (Kasukawa et al., 2011); it contains a genome-wide expression-profile of 51 regions. We exploited this publicly available expression-profile database to check our own gene-microarray analysis. Data appertaining to the lateral hypothalamus were compared with those derived from the cerebral cortex and the hippocampus.

### Metacore software analysis

Further analysis of the data was performed using MetaCore (Thomson Reuters, New York, NY, USA), a data mining and pathway-analysis software facility, which is commonly implemented for a functional analysis of gene-microarray findings. The known and putative functions of specific genes, as well as their involvement in biological and molecular processes, were also studied, using the GO (Gene Ontology) bioinformatics platform and the UniProtKB/Swiss-Prot database.

## Results

### Gene-expression analysis of the parvafox nucleus

Using Partek® Genomics Suite™ software, we first examined the expression levels of the genes that are known to be enriched in the parvafox nucleus, such as *Slc17a6* and *Pvalb*, and of those that have been demonstrated to remain unexpressed, such as *Slc17a7* and *Gad1*. This analysis confirmed the expression levels of *Slc17a6*, the gene encoding the vesicular glutamate transporter 2 (Vglut2), to be higher in the parvafox nucleus than in either the cortex or the hippocampus. *Pvalb* is expressed in both the cortex and the hippocampus, but at a much higher level in the parvafox nucleus. The expression levels of *Slc17a7* (encoding Vglut1) and *Gad1* (coding for the GABA-synthesizing enzyme glutamate decarboxylase 1) in the parvafox nucleus were low compared to those in the cortex and the hippocampus (not shown).

A further analysis of the data was conducted using the Bioconductor package limma in R. A gene was considered to be enriched in the parvafox nucleus if its expression levels in the mice were 2-fold higher than those in both the hippocampus and the cortex. This was a very strict selection criterion, and its investigation revealed the enrichment of 67 genes in the parvafox nucleus (Table 1). The ABA-ISH and the B^*^ database were screened for the specific expression of each of these genes in the region occupied by the parvafox nucleus. The function of these genes was ascertained by a search of the UniProtKB/Swiss-Prot database. The genes that were enriched in the parvafox nucleus (Table 1) can be classified as follows: neuropeptide activity (*Adcyap1, Npb, Nxph4, Pmch*), receptors (*Glra2, Gpr139, Vmn1r7*), transporters (*Slc17a6*), chloride channels (*Ano4*), enzymes (*Agxt2l1, Dgkk, Fam113b, Gpx3, Hyi, Phyhd1, Plcb4, Ucp2*), peptide-hormone precursors (*Agt*), transcription factors (*Ddx5, Ebf3, Pitx2*), peptidase inhibitors (*Itih3, Serpinb1b*), apolipoprotein (*Apoc1*), angiogenesis regulators (*Baiap3*), cytoskeleton growth regulator (*Capza1*), nucleosome-interacting proteins (*Hist1h1b, Hmgxb4*), cell recognition (*Igsf1*), cell-cell adhesion (*Fndc3a, Mpp7*), neural development (*Slitrk6*), cell growth and differentiation (*Sfrp5*), regulators of membrane traffic (*Rhobtb3, Yif1a*), unknown functions (*Lhfpl3, Lhfpl5, Mthfd2l, Rnf122, Trav9d*-3, *Zfp455, Zfp784*), non-coding RNA (*Rmst, Rny1, Snord42b*), and RNA-processing (*Scarna3a*). Eight predicted genes, 6 RIKEN-cDNAs and 7 non-characterized sequences were also enriched. We then expanded the initial list with 15 potential genes (*Cbln1, Drd2, Ephb1, Foxb1, Glra1, Glra3, Kcnab3, Lgi2, Lhx1, Lrrc6, Ndufs1, Npsr1, Penk, Vamp1, 5830454E08Rik*), which initially failed the selection criteria but appeared to be highly overexpressed in either the hippocampus or the cortex, or in one of the mice (Table 1), thus raising the total number of genes potentially expressed in the parvafox nucleus to 82.

**Table 1:** **List of the 82 genes that were enriched in the parvafox nucleus relative to the expression levels in the cortex and/or the hippocampus in our own gene-microarray analysis, and their localization counter-checked in the ABA-ISH database**.

**Gene**	**Complete name**	**fc_m355_nu_cx**	**fc_m355_nu_hp**	**fc_m511_nu_cx**	**fc_m511_nu_hp**	**NOT in ABA**	**NEG in ABA**	**POS in ABA**	**POS in LH**	**POS in parvafox**
**Adcyap1**	Adenylate cyclase activating polypeptide 1	3.92	27.23	5.00	4.81				v	v
**Agt**	Angiotensinogen	2.33	3.27	8.61	5.77				v	v (glia)
**Agxt2l1**	Alanine-glyoxylate aminotransferase 2-like 1	2.26	4.45	6.15	5.99				v	
**Ano4**	Anoctamin 4	2.12	2.83	3.20	2.81			v		
**Apoc1**	Apolipoprotein C-I	3.67	4.45	6.42	6.59				v	
**Baiap3**	BAI1-associated protein 3	2.21	3.72	2.86	3.57				v	
**Capza1**	Capping protein muscle Z-line, alpha 1	2.09	2.38	3.90	2.65				v	v
*Cbln1*	Cerebellin-1-precursor	4.51	4.17	2.68	1.74					v
**Ddx5**	DEAD (Asp-Glu-Ala-Asp) box polypeptide 5	2.85	3.42	2.04	7.50				v	v
**Dgkk**	Diacylglycerol kinase kappa	2.85	5.02	7.65	9.05				v	
*Drd2*	Dopamine 2 receptor	2.95	2.83	−4.95	2.29					v
**Ebf3**	Early B cell factor 3	7.46	6.31	5.49	6.33		v			
*Ephb1*	Ephrin b1 receptor tyrosine kinase	2.19	2.13	1.25	2.18					v
**Fam113b**	Family with sequence similarity 113, member B	2.46	2.33	2.61	2.54		v			
**Fndc3a**	Fibronectin type III domain containing 3A	2.65	2.55	2.60	2.55				v	v
*Foxb1*	Transcription factor	3.92	4.82	1.44	1.25		v			pos. in Literature
*Glra1*	Glycine receptor, alpha 1 subunit	−1.91	3.86	1.90	2.62					v
**Glra2**	Glycine receptor, alpha 2 subunit	3.11	6.32	3.71	4.98				v	v
*Glra3*	Glycine receptor, alpha 3 subunit	−1.19	10.41	21.52	16.93					v
**Gpr139**	G protein-coupled receptor 139	6.71	5.73	2.45	2.71		v			
**Gpx3**	Glutathione peroxidase 3	3.05	8.79	3.15	3.02				v	v
**Hist1h1b**	Histone cluster 1, H1b	5.18	4.42	2.89	24.43			v		
**Hmgxb4**	HMG box domain containing 4	2.09	2.27	2.27	2.23		v			
**Hyi**	Hydroxypyruvate isomerase homolog (E. coli)	4.04	2.87	3.98	2.18		v			
**Igsf1**	Immunoglobulin superfamily, member 1	2.89	2.90	14.59	13.69		v			
**Itih3**	Inter-alpha trypsin inhibitor, heavy chain 3	5.16	13.88	22.97	10.69				v	v (glia)
*Kcnab3*	Potassium channel, beta3, shaker type	2.09	2.44	1.43	−1.26					v
*Lgi2*	Leucine-rich g repeat	1.32	2.19	2.40	2.64					v
**Lhfpl3**	Lipoma HMGIC fusion partner-like 3	2.17	5.41	2.82	2.39		v			
**Lhfpl5**	Lipoma HMGIC fusion partner-like 5	3.90	5.19	4.99	2.27	v				
*Lhx1*	Lim-homobox protein 1	3.89	4.35	1.50	1.66					v
*Lrrc6*	Leucine rich repeat containing 6	−1.49	1.73	2.35	2.09					v
**Mpp7**	Membrane protein, palmitoylated 7	2.46	3.24	5.31	5.31			v		
**Mthfd2l**	Methylenetetrahydrofolate dehydrogenase 2-like	3.03	3.34	5.82	4.44		v			
*Ndufs1*	NADH dehydrogenase Fe-S protein 1	1.04	−1.35	2.03	3.18					v
**Npb**	Neuropeptide B	3.87	5.94	8.15	11.50		v			pos. in Literature
*Npsr1*	Neuropeptide S receptor	3.31	3.05	−4.50	1.20					v
**Nxph4**	Neurexophilin 4	6.02	14.85	4.93	2.23				v	v
*Penk*	Preproenkephalin	2.68	11.21	−5.37	4.01					v
**Phyhd1**	Phytanoyl-CoA dioxygenase domain containing 1	2.22	2.04	6.58	5.05	v				
**Pitx2**	Paired-like homeodomain transcription factor 2	3.06	2.83	6.61	5.78				v	v
**Plcb4**	Phospholipase C, beta 4	2.98	4.79	2.24	2.37			v	v	v
**Pmch**	Pro-melanin-concentrating hormone	66.57	137.92	85.36	115.62		v			
**Rhobtb3**	Rho-related BTB domain containing 3	2.71	3.10	2.39	2.14			v		
**Rmst**	Rhabdomyosarcoma 2 associated transcript	4.35	10.75	7.73	17.25	v				
**Rnf122**	Ring finger protein 122	2.12	2.31	3.41	2.18				v	v
**Rny1**	RNA, Y1 small cytoplasmic, Ro-associated	8.40	5.13	2.13	2.69	v				
**Scarna3a**	Small Cajal body-specific RNA 3A	4.03	5.56	4.08	5.62	v				
**Serpinb1b**	Serine peptidase inhibitor, clade B, member 1b	4.33	17.71	2.50	5.06			v		
**Sfrp5**	Secreted frizzled-related sequence protein 5	3.44	6.76	11.65	8.13			v		
**Slc17a6**	Solute carrier family 17, member 6	3.84	59.58	9.75	9.52				v	v
**Slitrk6**	SLIT and NTRK-like family, member 6	3.12	2.38	5.31	4.95			v		
**Snord42b**	Small nucleolar RNA, C/D box 42B	3.78	13.55	4.03	3.13	v				
**Trav9d-3**	T cell receptor alpha variable 9D-3	3.91	6.61	18.00	19.88	v				
**Ucp2**	Uncoupling protein 2	2.77	3.33	2.11	3.09			v		
*Vamp1*	vesicle associate membrane protein 1	−1.02	4.69	2.85	4.96					v
**Vmn1r7**	vomeronasal 1 receptor 7	2.08	2.58	2.01	2.51	v				
**Yif1a**	Yip1 interacting factor homolog A (S. cerevisiae)	2.43	2.08	2.20	2.22			v		
**Zfp455**	Zinc finger protein 455	3.18	3.78	2.31	2.13		v			
**Zfp784**	Zinc finger protein 784	5.04	2.87	2.29	2.06	v				
**1110015O18Rik**	RIKEN cDNA 1110015O18 gene	2.45	3.46	7.34	8.67	v				
**1700047M11Rik**	RIKEN cDNA 1700047M11 gene	3.85	3.47	5.37	12.00	v				
**3110083C13Rik**	RIKEN cDNA 3110083C13 gene	2.03	2.23	3.26	3.58	v				
**5031410I06Rik**	RIKEN cDNA 5031410I06 gene	2.09	2.91	4.10	6.81		v			
*5830454E08Rik*	RIKEN cDNA 5830454E08 gene	2.26	2.90	−1.69	1.38				v	
**A230001M10Rik**	RIKEN cDNA A230001M10 gene	2.23	3.38	2.11	3.48	v				
**B230323A14Rik**	RIKEN cDNA B230323A14 gene	4.47	5.23	6.74	6.73	v				
**Gm10408**	Predicted gene 10408	2.08	3.94	3.30	4.01	v				
**Gm10409**	Predicted gene 10409	2.08	4.36	3.95	2.51	v				
**Gm13157**	Predicted gene 13157	6.17	4.88	2.83	4.29	v				
**Gm19763**	Predicted gene, 19763	5.09	6.28	2.25	2.09	v				
**Gm3500**	Predicted gene 3500	2.01	2.66	2.45	2.55	v				
**Gm3515**	Predicted gene 3515	3.59	7.15	11.80	15.33	v				
**Gm410**	Predicted gene 410	2.22	2.61	2.91	2.73	v				
**Gm4988**	Predicted gene 4988	2.13	2.67	2.07	2.90		v			
**AffyID:17296943**	–	2.90	3.70	2.43	3.42	v				
**AffyID:17366828**	–	2.78	3.72	4.15	3.37	v				
**AffyID:17370057**	–	5.07	4.82	4.42	3.12	v				
**AffyID:17381937**	–	4.19	5.35	3.59	9.54	v				
**AffyID:17398719**	–	2.08	2.10	2.66	3.56	v				
**AffyID:17428186**	–	3.12	3.51	3.81	3.91	v				
**AffyID:17460252**	–	2.71	3.07	6.50	7.11	v				

*The enriched genes were selected on the basis of the fold-change values of each mouse represented in the 3rd to 6th column in the table (nu_cx, parvafox nucleus vs. cortex; nu_hp, parvafox nucleus vs. hippocampus). The gene-specific expression levels are represented by the log2 of the fluorescence intensity that was measured on the GeneChip®. The following possibilities were distinguished: absence from the ABA-database (NOT in ABA), negative results in the database (NEG in ABA), positive results in the database (POS in ABA), positive results in the database for the lateral hypothalamus (POS in LH), positive results in the database for the region that is occupied by the parvafox nucleus (POS in parvafox). The genes that are recognized for the first time to be enriched in the parvafox nucleus are indicated by a gray-shading of the background in the last column; those that were identified as being expressed in the parvafox nucleus in an earlier study by Girard et al. (2011) are not highlighted. Agt and Itih3 were exclusively expressed in glia (not in neurons). According to the ABA-ISH database, Npb and Foxb1 were not enriched in the hypothalamus, even though their expression in this region has been demonstrated by other authors, both immunohistochemically and by in-situ hybridization (Dun et al., 2005; Civelli et al., 2006)*.

(Genes that were found over-expressed compared to both the cortex and the hippocampus are formatted in bold, the genes over-expressed relative to either the cortex or the hippocampus and not both are in italic and underlined, and those that were found highly expressed in only one of the mice are in italic.)

By checking the expression of these 82 gene profiles in the ABA-ISH, their occurrence in the parvafox region of the lateral hypothalamus was confirmed visually for 26 (Table 1, last column), 10 of which were already known to occur in the parvafox region (Girard et al., 2011). Submitting these genes to an additional check with the B^*^ databases (Supplementary Table 1), this third criterion confirmed the enrichment of 13 of them (*Adcyap1, Agt, Cazpa1, Cbln1, Drd2, Fndc3a, Glra1, Glra2, Glra3, Gpx3, Itih3, Nxph4, Slc17a6*) in the parvafox nucleus. On the basis of morphological criteria in the ABA-ISH, we suspected that *Agt* and *Itih3* were expressed by glia rather than by neurons. Remarkably, both in the ABA-ISH and the B^*^ database, the results for neuropeptide b and *Foxb1* were negative (Table 1, Supplementary Table 1), despite they having been successfully identified by *in-situ* hybridization in the hypothalamus by other authors (Dun et al., 2005; Jackson et al., 2006; Schulz et al., 2007). Consequently, in these specific cases we interpret the ABA-ISH-database result as a false negative one.

For many of the other genes that were revealed by the microarray analysis to be enriched in the parvafox nucleus, corresponding confirmation in the ABA-ISH images or in the B^*^ database were either negative or not possible. For example, in the ABA-ISH the expression of 28 genes had not been performed and 14 genes were negative. Alternatively, the localization was either in the lateral hypothalamus at large (5 genes) or outside it (9 genes). In ABA-ISH images of sagittal sections, the parvafox nucleus was more difficult to identify with certainty, and the data were consequently less unequivocal. The findings of the detailed evaluation are summarized as a whole in the Table 1.

**Figure 1:**
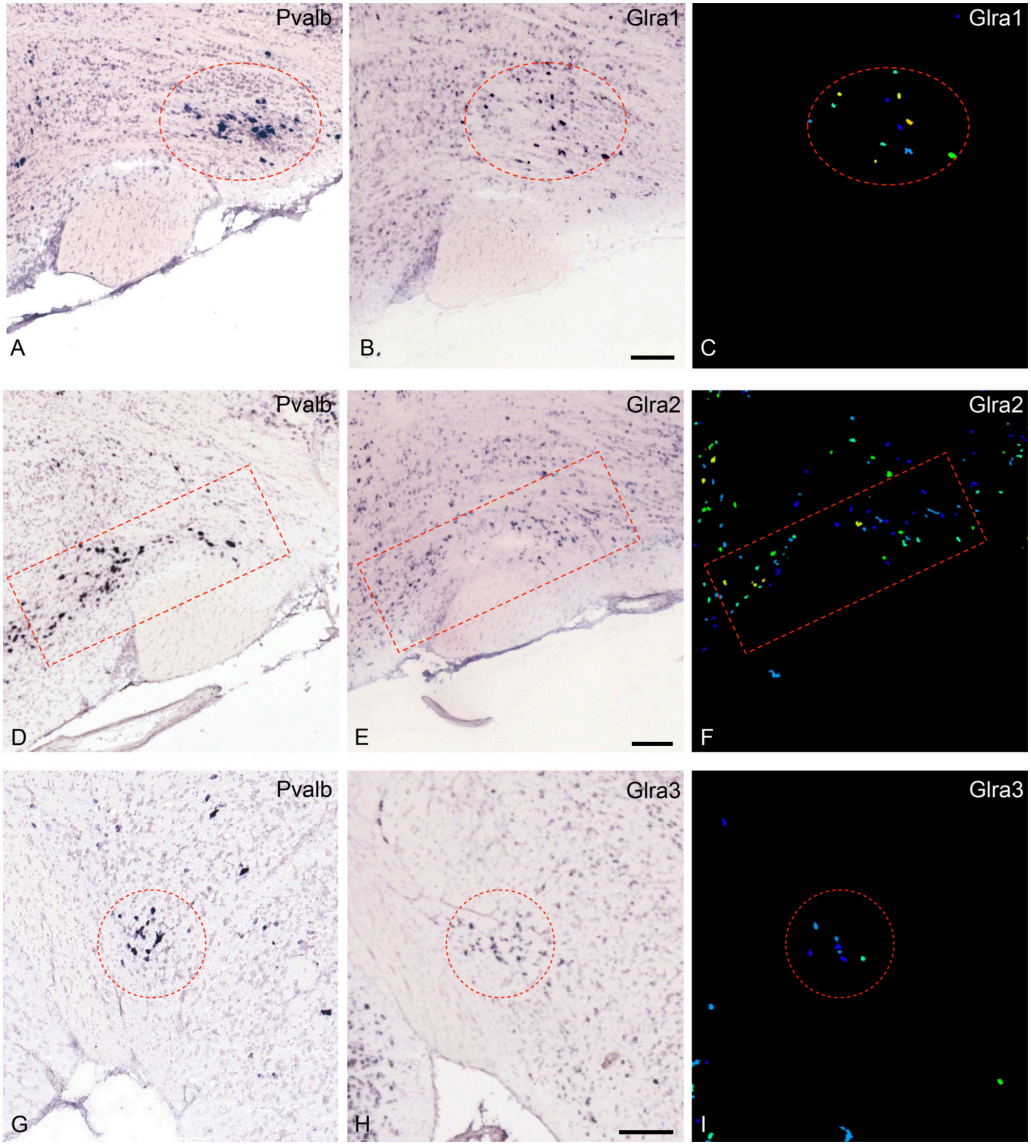
**Expression of the mRNA for the glycine-receptor subunits α-1, α-2 and α-3 in the parvafox nucleus as found in the ABA-ISH. (A,D,G)** *In situ* hybridization-images for *Pvalb*, downloaded from the ABA website, defining the location of the parvafox nucleus (surrounded by a red circle or rectangle). **(B,E,H)**: The mRNAs for the glycine-receptor subunits α-1, α-2, and α-3 are expressed within the confines of the parvafox nucleus. In **(C,F,I)** the expression level of Glra1, Glra2, and Glra3 is visualized with pseudocolors. **(A–F)** are sagittal sections and **(G–I)** coronal sections. (Image credit: Allen Institute.) Scale bars represent 100 μm.

**Figure 2:**
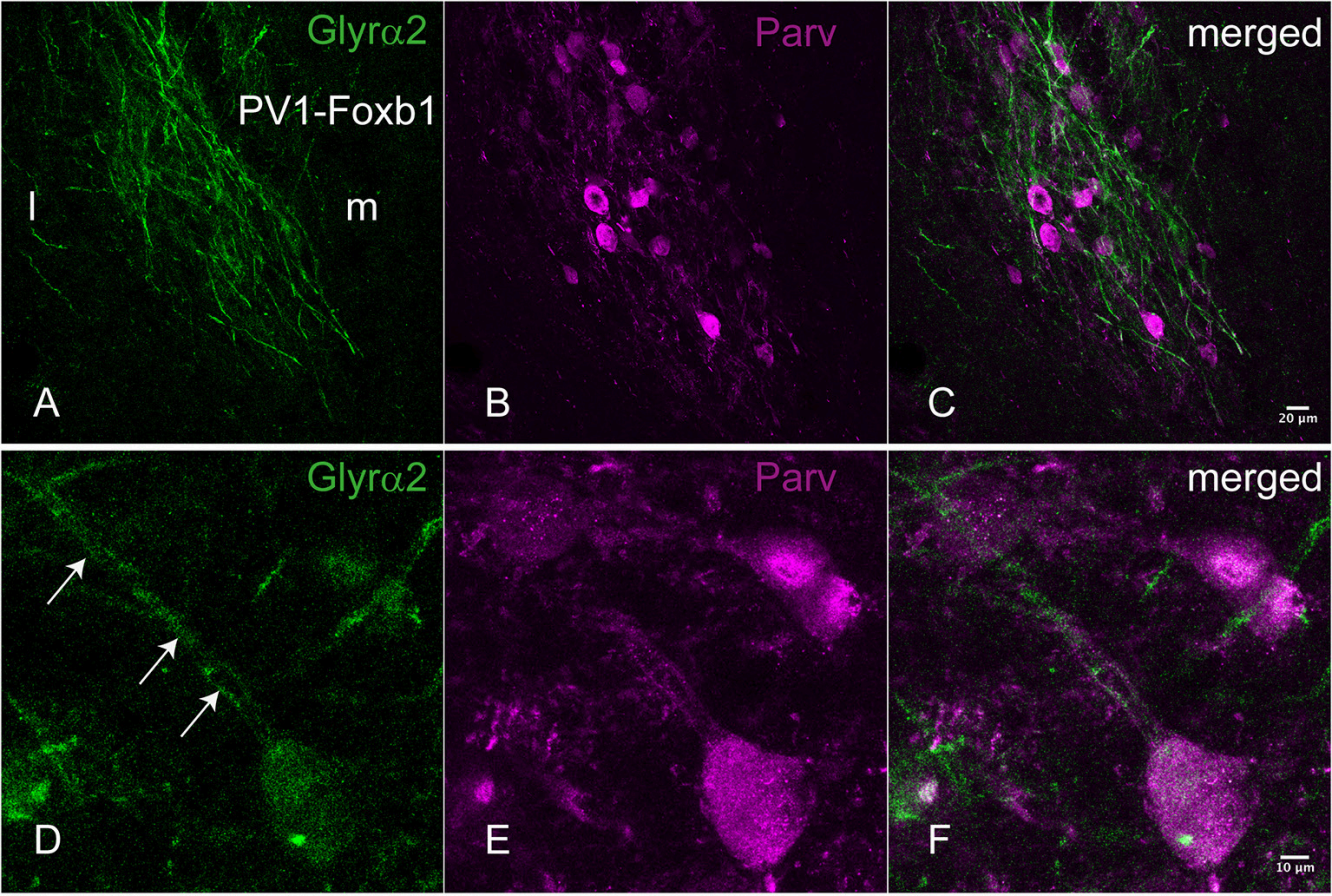
**Immunohistochemical localization of the Glyrα2 receptor in the parvafox nucleus**. In overview pictures, the Glyrα2 immunoreactive sites in the hypothalamus are concentrated in the ventrolateral hypothalamus and the mammillary nuclei. At low magnification, a meshwork of Glyrα2 immunoreactive fibers **(A)** is intermingled with Parv-immunoreactive neurons **(B,C)**. At higher magnification, Glyrα2 immunoreactivity decorates the dendrites (arrows, **D**) and cell body of Parv-positive neurons **(E,F)**.

The most noteworthy result was the finding that the glycine-receptors subunit alpha-2 (*Glra2*) was expressed at higher levels in the parvafox nucleus than in both the cortex and the hippocampus, with a mean fold-change of +4.53 (Table 1). The glycine-receptor subunit alpha-3 (*Glra3*), although excluded by our strict selection criteria, was also expressed at high levels in the parvafox nucleus, with a mean fold-change of +11.92 in mouse 511 (Table 1 and Figure 1B). Screening of the B^*^-microarray database revealed the glycine-receptor subunit alpha-1 (*Glra1*) to be likewise expressed at higher levels in the parvafox region (*FC* = 3.5) than in the hippocampus (see Supplementary Table 1). The expression of these three alpha-subunit isoforms of glycine receptors in the parvafox nucleus was confirmed by visual screening of the ABA-ISH database (Table 1, Figure 1) and for Glyrα2 additionally also by immunofluorescence staining with specific antibodies (Figure 2).

### Quantitative real-time PCR

Nine genes of particular interest because of their role as markers or neuromodulators (for the extended list of genes see Table 1) were selected to verify the results of the gene microarray study and to confirm their expression in the region of the parvafox nucleus by quantitative real-time PCR (Table 2). We calculated the expression values based on the 2^−ΔΔ*C*T^ method as described in the paper of Livak and Schmittgen (2001). The log_2_FC values obtained by the qRT-PCR assay are listed in Table 3. Student's *t*-tests were performed to assess statistical significance. We were able to confirm the enrichment of 9 genes in the parvafox nucleus: *Cbln1, Drd2, Ephb1, Foxb1, Glra1, Glra2, Glra3, Npb* and *Npsr1*. *P*-values calculated to compare the ΔC_T_ values of the parvafox nucleus to the cortex and hippocampus of both mice showed that the enrichment was significant for all nine genes: *Cbln1* (*p* = 0.0054) *Drd2* (*p* = 0.003), *Ephb1* (*p* = 0.00009), *Foxb1* (*p* = 0.0008), *Glra1* (*p* = 0.013), *Glra2* (*p* = 0.000001), *Glra3* (*p* = 0.003), *Npb* (*p* = 0.00073) and *Npsr1* (*p* = 0.007) (Table 3). These results coincide with the microarray fold change values; however, in some cases the expression levels were considerably higher when measured by qRT-PCR (see Supplementary Table 2).

**Table 2:** **A selection of 9 genes of particular interest because of their novelty and possible roles as markers or as neuromodulators (for an extended list of genes see Table 1) has been confirmed to be expressed in the transcriptome of the parvafox nucleus by qRT-PCR**.

	**Gene-array**	qRT PCR^*^	B^*^	**ABA**	**IC**	**Literature**
*Cbln1*	x	x	x	x		
*Drd2*	x	x	x	x		
*Ephb1*	x	x		x		
*Foxb1*	x	x		neg	x^**^	x
*Glra1*	x	x	x	x		
***Glra2***	**x**	**x**	**x**	**x**	**x**	
*Glra3*	x	x	x	x		
*Npb*	x	x		neg		x
*Npsr1*	x	x	No data	x		

We also examined the expression of these 9 genes in the BrainStars (B^*^) database and the ABA-ISH database, additionally we performed immunohistochemistry (IC) to validate the expression of Glra2 and Foxb1. Glra2 satisfies all five criteria for expression in the parvafox nucleus and represents the most robust result of this study. Cerebellin (Cbln1) and the dopamine 2 receptor (Drd2) meet four of the five criteria for occurrence in the parvafox nucleus.

Foxb1 and Npb are positive in our own gene-array and have been revealed by others (Dun et al., 2005; Civelli et al., 2006) to occur in the lateral hypothalamus, respectively in the parvafox nucleus (Bilella et al., 2014) but could not be confirmed neither in the ABA-ISH nor in the B^*^ databases.

* *p values calculated in Student's t-test (ΔCt of both nuclei vs. both cortex and hippocampus)*.

** *Positive in Foxb1-Cre-Egfp knock-in mice (Bilella et al., 2014)*.

**Table 3:** **Expression values presented as fold change on log_**2**_ scale obtained by qRT-PCR**.

	**Cbln1**	**Drd2**	**Ephb1**	**Foxb1**	**Glra1**	**Glra2**	**Glra3**	**Npb**	**Npsr1**
**log2FC NU vs. CX**									
AVG	4.03	1.89	4.21	1.96	3.07	1.23	2.29	4.62	1.67
SEM	0.86	0.54	0.57	0.25	2.30	0.32	0.45	0.45	0.62
*p* value	0.010618	0.034325	0.00611501	0.150336	0.156518	0.001574208	0.000616	0.003475	0.014121
**log2FC NU vs. HP**									
AVG	4.79	4.63	4.47	6.69	6.64	2.23	9.33	11.37	3.79
SEM	2.72	0.71	0.59	0.93	1.20	0.15	0.98	1.70	0.97
*p* value	0.034305	0.00072	1.0328E-05	2.47E-06	0.000141	4.03646E-13	4.79E-06	0.000126	0.003092
**log2FC NU vs. CX** + **HP**									
AVG	4.80	1.53	3.86	4.62	1.50	1.30	2.57	6.76	0.92
*p* value	0.005454	0.003403	9.0766E-05	0.000799	0.012946	6.00367E-07	0.003527	0.000728	0.007576
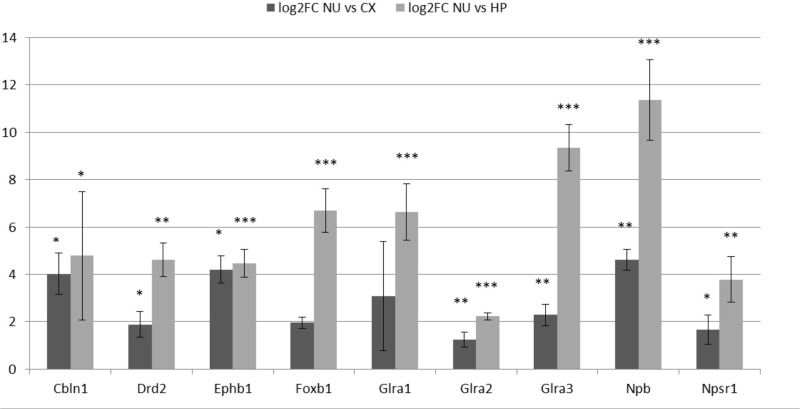									

Expression values were calculated based on the 2^−ΔΔCT^ method and then averaged. ΔC_T_ values of the parvafox nucleus were contrasted to ΔC_T_ values of the cortex, respectively to the ΔC_T_ values of the hippocampus; additionally, expression values were also calculated based on the ΔC_T_ values of the nucleus versus cortex AND hippocampus. When compared to the cortex, or to the hippocampus, the majority of the genes were significantly over-expressed in the parvafox nucleus, with exception of Foxb1 and Glra1 (compared to cortex). When compared to the cortex AND hippocampus, all the genes were significantly over-expressed in the parvafox nucleus. Error bars show SEM. (^*^p < 0.05, ^**^p < 0.005, ^***^p < 0.0005).

### Analysis of the putative roles of the genes that were enriched in the parvafox nucleus

We conducted a MetaCore (GeneGO)-enrichment analysis on a set of genes that had been revealed to be expressed in the parvafox nucleus by the combined results of the gene-microarray, the ABA-ISH database and the B^*^-database evaluations, as well as previous observations (Girard et al., 2011) (Table 4). This analysis revealed high expression levels of specific genes encoding proteins of various biological processes, the most noteworthy being synaptic transmission (Ionotropic glutamate receptor, Kv1.2, GluR5, Galpha(i)-specific amine GPCRs, GlyRA3, Galpha(q)-specific peptide GPCRs, HCN, KCNAB3, Cerebellin 1, NEFL, Kainate receptor, HCN2, VAMP1, Kv3.1, GlyRA1, PLC-beta), neuropeptide signaling pathway (Galpha(s)-specific CRF GPCRs, PACAP, GlyRA2, Neurexophilin 4, Galpha(q)-specific peptide GPCRs, Neuropeptide S receptor, CRHR1, Enkephalin A, GlyRA1), and regulation of the inhibitory postsynaptic membrane potential (Ionotropic glutamate receptor, GluR5, Galpha(i)-specific amine GPCRs, Kainate receptor, GlyRA1). A relation to behavioral response to pain was also manifested (Ionotropic glutamate receptor, Galpha(s)-specific CRF, GPCRs, GluR5, Galpha(q)-specific peptide GPCRs, CRHR1, Kainate receptor) (Table 4).

**Table 4:** A GeneGO (MetaCore) enrichment analysis was performed on 45 genes found enriched in our microarray, in the ABA-ISH and in the B^*^ database, supplemented with genes which had previously been found to be positive in the parvafox nucleus (Girard et al., 2011).

**Rank**	**GO Processes**	**Total**	***P*** **value**	**Min FDR**	**In Data**	**Network objects**
1	Synaptic transmission	826	3.628E-14	3.813E-11	17	KCNAB3, Ionotropic glutamate receptor, NEFL, Cerebellin 1, Kainate receptor, Kv1.2, GluR5, HCN2, VAMP1, Kv3.1, Galpha(i)-specific amine GPCRs, GLyRA3, GlyRA1, Galpha(q)-specific peptide GPCRs, PLC-beta, HCN
2	Neuropeptide signaling pathway	138	2.343E-12	1.231E-09	10	Galpha(s)-specific CRF GPCRs, PACAP, Enkephalin A, GlyRA2, Neurexophilin 4, GlyRA1, Galpha(q)-specific peptide GPCRs, Neuropeptide S receptor, CRHR1
3	Regulation of inhibitory postsynaptic membrane potential	24	2.331E-11	8.165E-09	6	Ionotropic glutamate receptor, Kainate receptor, GluR5, Galpha(i)-specific amine GPCRs, GlyRA1
4	Regulation of membrane potential	413	8.292E-11	2.179E-08	8	Ionotropic glutamate receptor, Kainate receptor, GluR5, HCN2, GlyRA1, Galpha(q)-specific peptide GPCRs, HCN
5	Behavioral response to pain	30	1.352E-10	2.842E-08	6	Ionotropic glutamate receptor, Kainate receptor, Galpha(s)-specific CRF GPCRs, GluR5, Galpha(q)-specific peptide GPCRs, CRHR1
6	Negative regulation of voltage-gated calcium channel activity	6	6.228E-10	1.091E-07	4	Galpha(s)-specific CRF GPCRs, Dopamine D2 receptor, Galpha(i)-specific amine GPCRs, CRHR1
7	Negative regulation of synaptic transmission, glutamatergic	19	1.140E-09	1.712E-07	5	Ionotropic glutamate receptor, Kainate receptor, GluR5, Dopamine D2 receptor, Galpha(i)-specific amine GPCRs
8	Startle response	41	4.127E-09	5.422E-07	5	Enkephalin A, Dopamine D2 receptor, Galpha(i)-specific amine GPCRs, GlyRA1
9	Regulation of JNK cascade	178	7.792E-09	9.099E-07	5	Ionotropic glutamate receptor, Kainate receptor, Ephrin-B receptors, Galpha(q)-specific peptide GPCRs, Ephrin-B receptor 1
10	Ion transport	1408	1.008E-08	1.059E-06	14	KCNAB3, Ionotropic glutamate receptor, Kainate receptor, Kv1.2, GluR5, GlyRA2, HCN2, SLC17A6, SCN4B, Kv3.1, GlyRA3, GlyRA1, HCN

*The distribution in GeneGO Processes revealed enrichment in pathways and processes involved in subcellular mechanisms (neurotransmission, ion transport, neuropeptide signaling), as well as in the startle response and the behavioral response to pain*.

### Immunohistochemistry revealed Parv-positive neurons of the parvafox nucleus to express glycine receptor α2

**Figure 3:**
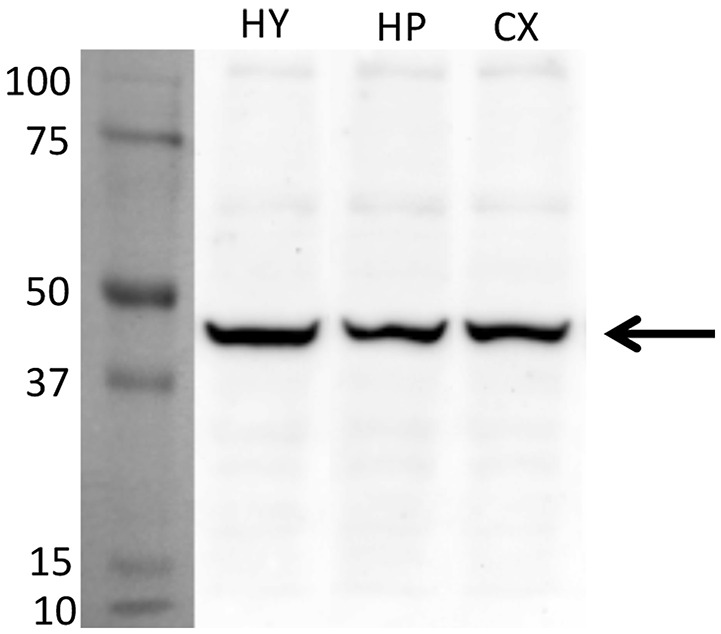
**Detection of Glyrα2 on hypothalamic, hippocampal and cortical extracts by immunoblot**. The antiserum against Glyrα2 detects a single band below 50 KDa (arrow) in an immunoblot of hypothalamic, hippocampal and cerebral cortical extracts submitted to SDS-gel electrophoresis. (HY, hypothalamus; HP, hippocampus; CX, cortex).

For an additional independent verification of the most robust and interesting results, an antiserum against the Glyrα2-receptor was used to detect immunoreactivity in the hypothalamus. Positive immunolabeling was selectively observed in a few hypothalamic regions, one being the parvafox nucleus (Figures 2A–C). At higher magnification, the immunoreactivity was revealed to be associated with the plasma membrane of the Parv-positive neurons in this region (Figures 2D–F). Immunoblot analysis of murine hypothalamus protein extract with the antiserum against Glyrα2 resulted in a sharp band below 50 KDa, the expected molecular weight for the Glyrα2 receptor (Figure 3), confirming the presence of Glyrα2 in the hypothalamus, as well as the specificity of the Glyrα2-antibody used in our study. In addition to the hypothalamus, we detected Glyrα2 in hippocampal and cortical lysates (Figure 3) as well, regions that served as reference structures in the microarray analysis.

### GlyT2-EGFP-positive axon terminals end on Parv-neurons of the parvafox nucleus

**Figure 4:**
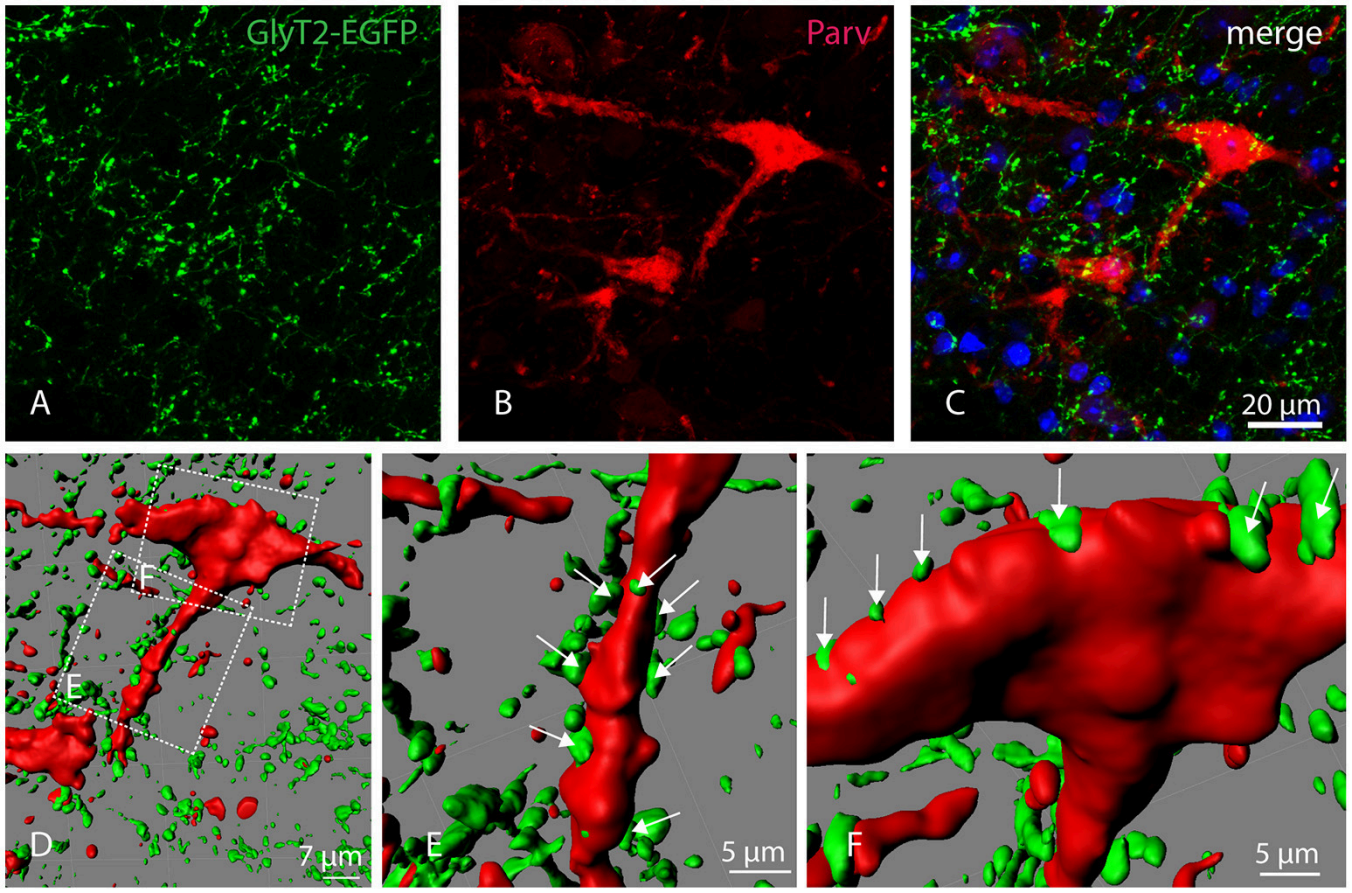
**Localization of GlyT2-EGFP-positive axons and axon terminals in the parvafox nucleus**. Confocal laser micrographs of double-immunohistochemistry for GFP (green, **A**) and Parv (red, **B**) performed on brain sections of transgenic GlyT2-EGFP mice revealed the presence of several axons and axon terminals in the hypothalamic region encompassing the parvafox nucleus. The merged image **(C)** shows GlyT2-EGFP-positive terminals around the perikaryon and the dendrites of a Parv-positive neuron. Three-dimensional reconstruction by Imaris **(D)** reveals several EGFP-positive terminals on the dendrite (**E**, see arrows), as well as on the cell body (**F**, see arrows) of the Parv-neuron.

To confirm that Parv-neurons of the parvafox-nucleus indeed receive input from glycinergic nerve cells, Glycine transporter 2 (GlyT2)-expression was studied in GlyT2-EGFP mice (Zeilhofer et al., 2005). Double-immunolabeling of Parv and GFP revealed a dense net of GlyT2-EGFP positive axons and axon terminals in the parvafox region. We found that several GlyT2-EGFP axon terminals indeed ended on Parv-neurons (Figures 4A–C). Confocal laser microscope z-stacks were then subjected to 3D-reconstruction with the Imaris software for a better visualization (Figure 4D). The 3D-reconstruction demonstrated GlyT2-EGFP axon terminals in apposition both to the dendrites (Figure 4E, see arrows) and to the perikarya (Figure 4F, see arrows) of Parv-neurons of the parvafox nucleus.

## Discussion

Laser-capture microdissection has been used in a variety of publications for the identification of marker genes that may help in the genetic dissection of functionally well characterized regions of the medial hypothalamus (Segal et al., 2005; Paulsen et al., 2009; Landmann et al., 2012; Lee et al., 2012; Humerick et al., 2013).

The aim of our gene-microarray analysis was primarily the search for neuromodulator genes in neurons of an ill-defined lateral hypothalamic region of as yet unknown function. Notwithstanding the methodological complexity, the expression of the receptors for the inhibitory neurotransmitter glycine in the parvafox nucleus is now well documented by our study.

This study has permitted an extension of the list of genes that are preferentially expressed in the parvafox nucleus of the ventrolateral hypothalamus. In addition to the 76 genes that were previously identified to be expressed by Girard et al. (2011), additional 82 (some overlapping) have been shown to be enriched in this region by our gene-microarray analysis. A survey of the ABA-ISH and B^*^ databases for their hypothalamic distribution confirmed the expression of at least 13 of these genes in the parvafox nucleus. The expression of nine particularly relevant genes involved in neuromodulation was corroborated by qRT-PCR.

Interestingly, only a few genes (*Adcyap1, Pitx2, Slc17A6, Plb4, Itih3, Hist1h1b, Tmem163*) were revealed to be commonly expressed by the gene-microarray analysis and the ABA-ISH-database screening (Girard et al., 2011) thereby indicating that the two methodologies differ in sensitivity, specificity and selectivity. Although, both the *in-situ*-hybridization technique and the gene-microarray approach revealed an expression of various gene-families (e.g., neurotransmitters, transcription factors, extracellular molecules), the individual members thereof that were revealed by the ABA-ISH-database screening differed from those that were brought to light by the gene-microarray analysis.

The most obvious explanation of this discrepancy is that the gene-array search for the relative abundances based on a comparison between regions, namely, between the hypothalamus and either the cortex or the hippocampus. Consequently, the genes that are expressed in both regions were excluded. For example, the potassium channels (*Kcna1, Kcna2, Kcnab2, Kcnab3, Kcnc1, Kcnc2*, and *Kcnk1*), which are responsible for the high firing rates of Parv-positive neurons in the cortex and the hippocampus, were recognized as being expressed in the parvafox nucleus by the ABA-ISH-database screening (Girard et al., 2011) but not by the gene-microarray analysis (Table 1). Nevertheless, a detailed comparison of the long list of expressed genes in the gene-microarrays of both mice revealed that approximately 40% of those that were registered in the study of (Girard et al., 2011) were also enriched in the gene-microarray of the parvafox nucleus (results not shown).

For some genes, no *in-situ*-hybridization data exist as yet in the ABA-ISH (Table 1). For others (e.g., *Npb, Foxb1*), screening of the ABA-ISH database yielded negative results, even though the expression of such genes in the hypothalamus has been demonstrated by *in-situ* hybridization or immunohistochemistry in other reports (Dun et al., 2005; Civelli et al., 2006). Low-level transcripts may be below the threshold of detection by *in situ* hybridization techniques, and more sensitive techniques such as qRT-PCR may be needed to validate their expression in the parvafox nucleus. Hence, falsely negative results partially account for the discrepant findings.

On the other hand, it is also conceivable that the gene-array technique yields falsely-positive or falsely-negative results (Mitchell and Mirnics, 2012). The former may arise from the non-specific binding of cDNAs to homologous oligonucleotide probes, and the latter from the privileged amplification of certain mRNAs at the expense of others.

Hence, taking into account the combined results of the three approaches (Gene-array/ABA/B^*^) maximizes the reliability of the information we have gained. The counterchecking of findings revealed by a gene-microarray analysis against those disclosed by *in-situ* hybridization (screening of the ABA-ISH database) and in the B^*^ database, is a legitimate mean of predicting the expression profiles of the neurons in the parvafox nucleus. Albeit so, it represents only a first approximation: the definitive allocation of gene-expression activity to a specific sub-population of neurons awaits the instigation of antibodies against the corresponding proteins for a definitive immunohistological confirmation of their localization to specific sub-population of neurons (Parv- or Foxb1-expressing). Furthermore, functional *in-vitro* studies investigating for example the effects of blocking glycinergic transmission or applying locally the corresponding neurotransmitter will be needed to confirm the physiological roles of the genes of interest.

The glycinergic inhibitory system is particularly powerful in the spinal cord and the brainstem, where virtually all immunoreactive cell-bodies are located (Kirsch, 2006). However, glycine-immunoreactive fibers and terminals have been observed in various parts of the brain by different authors (Zeilhofer et al., 2005; Barreiro-Iglesias et al., 2013) and glycine-induced chloride-currents were discovered already in 1989 in dissociated hypothalamic neurons (Akaike and Kaneda, 1989). Functional glycine receptors have been detected in higher brain centers, such as the hippocampus (Brackmann et al., 2004; Keck and White, 2009; Xu and Gong, 2010), and the glycine transporter 2 has been demonstrated in the whole brain as well as in the hypothalamus (Zeilhofer et al., 2005). The glycine receptor alpha 2 subunit alone can build functional receptors in adults and is particularly expressed in hippocampus and cortex (Meier et al., 2014). The expression of other components of the glycinergic system (glycine itself, glycine transporters, Glyrα1 and Glyrα3 glycine receptors, gephyrin) in the parvafox nucleus has been confirmed partly by the ABA-ISH-database screening (Figure 1) and by ongoing immunohistochemical studies. Hence, glycine appears to be an important inhibitory neurotransmitter in the parvafox nucleus, as also underlined by the Gene-GO enrichment analysis. The cell bodies giving rise to the axonal endings that abut on the neurons of the parvafox nucleus may be located in the brainstem, where aggregates of glycinergic neurons have been described (Zeilhofer et al., 2005).

In conclusion, the use of the laser capture microdissection and gene-microarray-based approach in combination with data verification in the ABA-ISH and B^*^ databases has proved to be on efficient means of gaining further insight into the transcriptome of the murine parvafox nucleus. Nine genes were confirmed by independent techniques to be enriched therein. Some of these markers may be useful to define subpopulations of parvafox neurons as a prelude to studying their circuitry and function. The augmented expression levels of several of these genes shed light on the functional mechanisms that may be operative in the parvafox nucleus, for example, the glycine-mediated inhibitory control.

## Author contributions

VS and GWA contributed equally to this work. VS and GWA designed and conducted the experiments, analyzed and interpreted results, and wrote the paper. MRC designed the experiments and wrote the paper.

### Conflict of interest statement

The authors declare that the research was conducted in the absence of any commercial or financial relationships that could be construed as a potential conflict of interest.

## Abbreviations

3V: third ventricle
ABA: Allen Brain Atlas
ABA-ISH: Allen Brain Atlas of *in-situ* hybridizations
BMA: basomedial amygdalar nucleus
Cx: cortex
DMH: dorsomedial hypothalamic nucleus
f: fornix
fc: fold-change
*Gad1*: glutamate decarboxylase 1
*Glra1*, glycine receptor: alpha 1 subunit (gene)
*Glra2*, glycine receptor: alpha 2 subunit (gene)
*Glra3*, glycine receptor: alpha 3 subunit (gene)
Glyrα2, glycine receptor: alpha 2 subunit (protein)
Glyrα3, glycine receptor: alpha 3 subunit (protein)
hp: hippocampus
ic: internal capsule
ISH: *in-situ* hybridization
LHA: lateral hypothalamic area
MEA: medial amygdalar nucleus
mt: mammillothalamic tract
ns: nigrostriatal bundle
opt: optic tract
Parv: parvalbumin (protein)
*Pvalb*: parvalbumin (gene)
*Slc17a6*: solute carrier family 17 member 6
*Slc17a7*: solute carrier family 17 member 7
STh: subthalamic nucleus
TH: thalamus
VMH: ventromedial hypothalamic nucleus
ZI: Zona incerta.
