# Supplementary material for: Parvalbumin-Neurons of the Ventrolateral Hypothalamic Parvafox Nucleus Receive a Glycinergic Input: A Gene-Microarray Study
Source: Front Mol Neurosci. 2017 Jan 23;10:8. doi: 10.3389/fnmol.2017.00008 (PMC5253383; doi:10.3389/fnmol.2017.00008)
Supplement: Supplementary file 1 [file Table1.DOCX]

Supplementary Material

**Parvalbumin-neurons of the ventrolateral hypothalamic parvafox nucleus receive glycinergic input: a gene-microarray study**

**Viktória Szabolcsi^1 #^, Gioele W. Albisetti^1 #^, Marco R. Celio^1^ ***

^1^ Anatomy and Program in Neuroscience, Department of Medicine, University of Fribourg, Rte. A. Gockel 1, CH-1700 Fribourg, Switzerland

^#^ These authors contributed equally to this work.

* **Correspondence** should be sent to: [marco.celio@unifr.ch](mailto:marco.celio@unifr.ch)

**Supplementary Table 1**

| Gene | Gene ID | FC LH vs CX | p value LH vs CX | FC LH vs HP | p value LH vs HP | p value LH vs CX AND HP |
| --- | --- | --- | --- | --- | --- | --- |
| **Adcyap1** | 1423427_at | 1.24 | 0.0726834 | 2.46 | **0.0004036** | **0.018887424** |
| **Agt** | 1423396_at | 7.86 | **0.0010504** | 5.42 | **7.664E-06** | **4.3754E-06** |
| **Capza1** | 1439455_x_at | 2.86 | **0.0106857** | 2.55 | **4.443E-05** | **4.96293E-06** |
| **Cbln1** | 1423286_at | 7.14 | **0.0070729** | 6.22 | **5.044E-06** | **4.64485E-07** |
| Ddx5 | 1419653_a_at | 1.05 | 0.155772 | -1.13 | 0.5626933 | 0.850029711 |
| **Drd2** | 1418950_at | 1.83 | 0.0653481 | 1.57 | **0.0267202** | **0.008156468** |
| Ephb1 | 1455188_at | 1.19 | 0.1459404 | 1.34 | 0.1559188 | 0.132049607 |
| **Fndc3a** | 1426903_at | 1.52 | **0.0233157** | 1.65 | **3.201E-05** | **5.68854E-06** |
| Foxb1 | 1420705_at | -1.03 | 0.727331 | 1.10 | 0.1657093 | 0.377286229 |
| **Glra1** | 1437139_at | 3.44 | **0.019975** | 3.50 | **9.655E-06** | **0.000234375** |
| **Glra2** | 1434098_at | 11.17 | **0.0055903** | 13.03 | **4.234E-05** | **2.63536E-06** |
| **Glra3** | 1450239_at | 1.76 | 0.0533409 | 2.12 | **0.0009442** | **0.000460168** |
| **Gpx3** | 1449106_at | 7.47 | **0.0041664** | 10.07 | **3.202E-06** | **6.54837E-07** |
| **Itih3** | 1449123_at | 9.51 | **0.0046314** | 12.69 | **1.201E-06** | **2.53953E-07** |
| Lrrc6 | 1440798_x_at | -1.11 | 0.9583268 | -1.05 | 0.9166524 | 0.054870038 |
| Ndufs1 | 1425143_a_at | 1.07 | 0.6015425 | 1.05 | 0.5330418 | 0.410063697 |
| Npb | 1440240_at | 1.07 | 0.5305209 | 1.02 | 0.813478 | 0.69778449 |
| Npsr1 | no data |  |  |  |  |  |
| Rnf122 | 1447101_at | 1.09 | 0.4808282 | 1.05 | 0.9450077 | 0.804172849 |
| **Nxph4** | 1429753_at | 1.33 | **0.0402779** | 1.47 | **0.0002628** | **0.000226711** |
| Pitx2 | 1424797_a_at | -1.08 | 0.3188974 | -1.08 | 0.3400413 | 0.281844749 |
| Plcb4 | 1425338_at | 1.08 | 0.9061606 | 2.23 | **0.000971** | 0.081341598 |
| **Slc17a6** | 1418610_at | 14.73 | **0.0011705** | 12.76 | **0.0014281** | **0.000182213** |

**Legends**

**Supplementary Table 1**:

Counterchecking the expression of potentially relevant genes found in our gene array with results of the B* database (GEO Accession Series GSE16496). The 13 genes in bold are enriched in the lateral hypothalamus (LH) as compared to cortex (CX) and hippocampus (HP) at p<0.05. (Gene IDs represent the Affymetrix Probe Set ID on the GPL1261 Platform.)

**
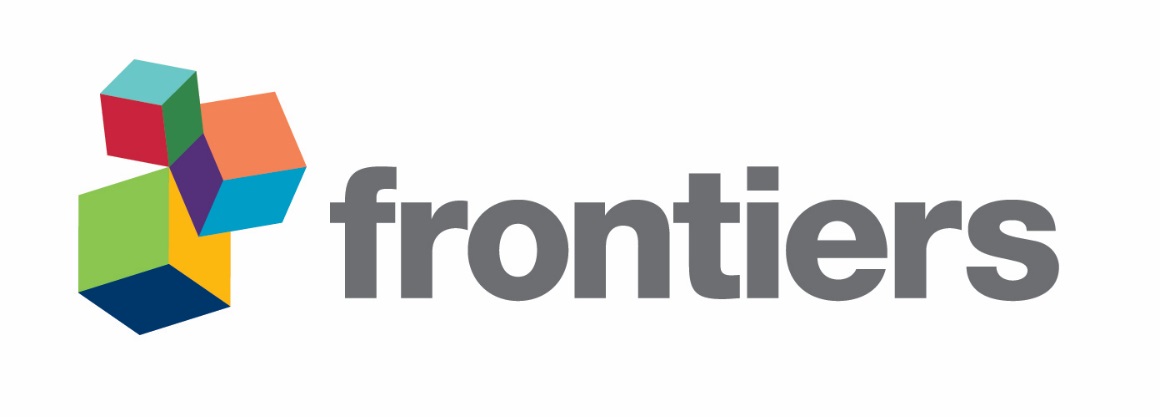
**
