# Supplementary material for: Parvalbumin-Neurons of the Ventrolateral Hypothalamic Parvafox Nucleus Receive a Glycinergic Input: A Gene-Microarray Study
Source: Front Mol Neurosci. 2017 Jan 23;10:8. doi: 10.3389/fnmol.2017.00008 (PMC5253383; doi:10.3389/fnmol.2017.00008)
Supplement: Supplementary file 2 [file Table2.DOCX]

Supplementary Material

**Parvalbumin-neurons of the ventrolateral hypothalamic parvafox nucleus receive glycinergic input: a gene-microarray study**

**Viktória Szabolcsi^1 #^, Gioele W. Albisetti^1 #^, Marco R. Celio^1^ ***

^1^ Anatomy and Program in Neuroscience, Department of Medicine, University of Fribourg, Rte. A. Gockel 1, CH-1700 Fribourg, Switzerland

^#^ These authors contributed equally to this work.

* **Correspondence** should be sent to: [marco.celio@unifr.ch](mailto:marco.celio@unifr.ch)

**Supplementary Table 2**

| m355 log2FC | NU vs CX, microarray | NU vs CX, qRT-PCR | NU vs HP, microarray | NU vs HP, qRT-PCR |  | m511 log2FC | NU vs CX, microarray | NU vs CX, qRT-PCR | NU vs HP, microarray | NU vs HP, qRT-PCR |
| --- | --- | --- | --- | --- | --- | --- | --- | --- | --- | --- |
| Cbln1 | 2.17 | 5.93 | 2.06 | 10.86 |  | Cbln1 | 1.42 | 2.13 | 0.80 | -1.29 |
| Drd2 | 1.56 | 2.49 | 1.50 | 7.16 |  | Drd2 | -2.31 | 0.46 | 1.20 | 2.33 |
| Ephb1 | 1.13 | 5.47 | 1.09 | 3.25 |  | Ephb1 | 0.33 | 2.95 | 1.12 | 5.69 |
| Foxb1 | 1.97 | 2.40 | 2.27 | 9.00 |  | Foxb1 | 0.52 | 1.28 | 0.32 | 4.15 |
| Glra1 | -0.94 | -1.77 | 1.95 | 5.21 |  | Glra1 | 0.92 | 7.91 | 1.39 | 8.07 |
| Glra2 | 1.64 | 0.28 | 2.66 | 2.17 |  | Glra2 | 1.89 | 2.19 | 2.32 | 2.30 |
| Glra3 | -0.26 | 1.74 | 3.38 | 10.88 |  | Glra3 | 4.43 | 2.84 | 4.08 | 7.79 |
| Npb | 1.95 | 4.76 | 2.57 | 8.93 |  | Npb | 3.03 | 5.02 | 3.52 | 16.94 |
| Npsr1 | 1.73 | 2.21 | 1.61 | 5.94 |  | Npsr1 | -2.17 | 1.13 | 0.26 | 1.64 |
|  |  |  |  |  |  |  |  |  |  |  |
| 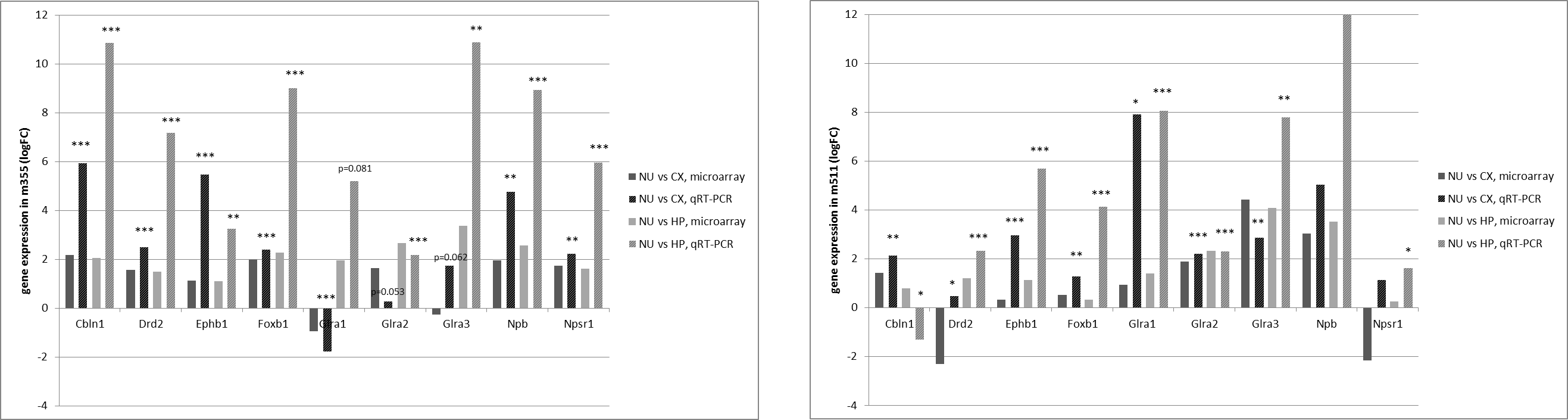   \|  \| \| --- \| |  |  |  |  |  |  |  |  |  |  |
|  |  |  |  |  |  |  |  |  |  |  |
|  |  |  |  |  |  |  |  |  |  |  |
|  |  |  |  |  |  |  |  |  |  |  |
|  |  |  |  |  |  |  |  |  |  |  |
|  |  |  |  |  |  |  |  |  |  |  |
|  |  |  |  |  |  |  |  |  |  |  |
|  |  |  |  |  |  |  |  |  |  |  |
|  |  |  |  |  |  |  |  |  |  |  |
|  |  |  |  |  |  |  |  |  |  |  |
|  |  |  |  |  |  |  |  |  |  |  |
|  |  |  |  |  |  |  |  |  |  |  |
|  |  |  |  |  |  |  |  |  |  |  |
|  |  |  |  |  |  |  |  |  |  |  |
| \| 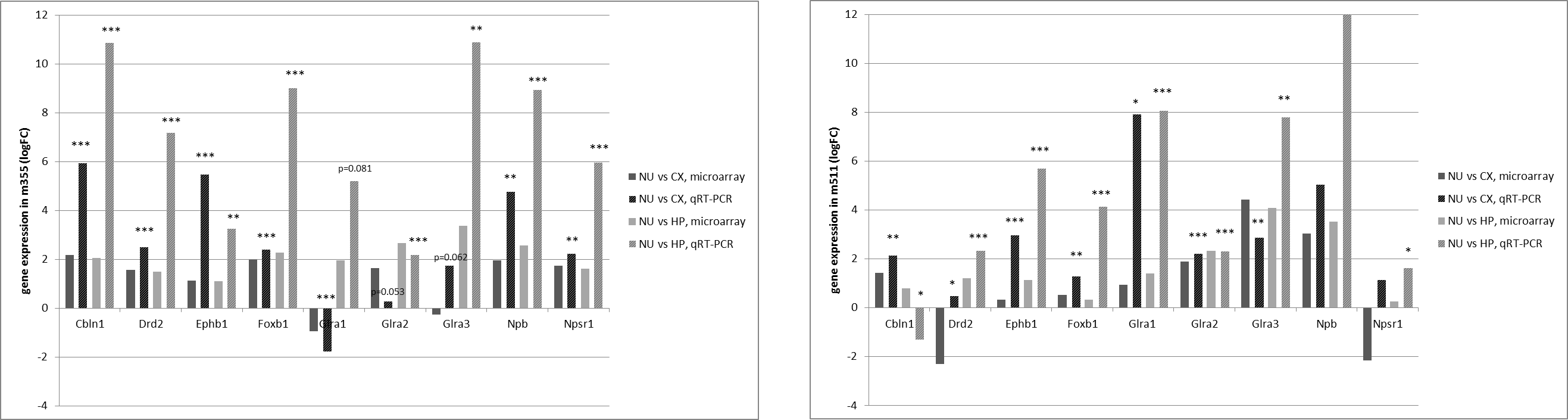 \| \| --- \| |  |  |  |  |  |  |  |  |  |  |
|  |  |  |  |  |  |  |  |  |  |  |
|  |  |  |  |  |  |  |  |  |  |  |
|  |  |  |  |  |  |  |  |  |  |  |
|  |  |  |  |  |  |  |  |  |  |  |
|  |  |  |  |  |  |  |  |  |  |  |
|  |  |  |  |  |  |  |  |  |  |  |
|  |  |  |  |  |  |  |  |  |  |  |
|  |  |  |  |  |  |  |  |  |  |  |
|  |  |  |  |  |  |  |  |  |  |  |
|  |  |  |  |  |  |  |  |  |  |  |

**Legends**

**Supplementary Table 2**:

Validation of the gene microarray results by qRT-PCR. Expression values are presented as fold change on log_2_ scale. Expression values of the parvafox nucleus obtained by qRT-PCR are compared to the expression values obtained by gene microarray in each mouse (m355 and m511) and presented as log_2_FC nucleus versus cortex, respectively log_2_FC nucleus versus hippocampus. Student’s t-tests were performed to assess statistical significance of the expression values obtained by qRT-PCR. (** p<0.05, ** p<0.005, *** p<0.0005*)

**
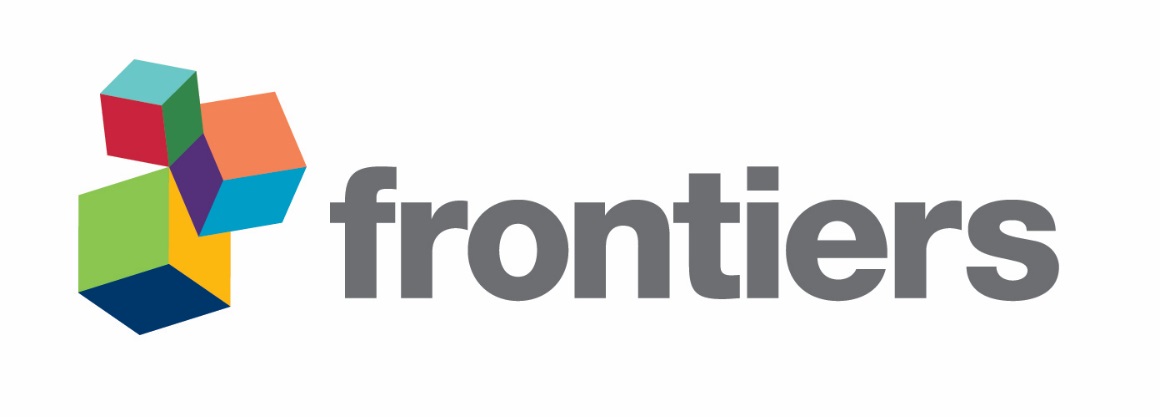
**
