# Supplementary material for: Parvalbumin-Neurons of the Ventrolateral Hypothalamic Parvafox Nucleus Receive a Glycinergic Input: A Gene-Microarray Study
Source: Front Mol Neurosci. 2017 Jan 23;10:8. doi: 10.3389/fnmol.2017.00008 (PMC5253383; doi:10.3389/fnmol.2017.00008)
Supplement: Supplementary file 3 [file Table3.DOCX]

Supplementary Material

**Parvalbumin-neurons of the ventrolateral hypothalamic parvafox nucleus receive glycinergic input: a gene-microarray study**

**Viktória Szabolcsi^1 #^, Gioele W. Albisetti^1 #^, Marco R. Celio^1^ ***

^1^ Anatomy and Program in Neuroscience, Department of Medicine, University of Fribourg, Rte. A. Gockel 1, CH-1700 Fribourg, Switzerland

^#^ These authors contributed equally to this work.

* **Correspondence** should be sent to: [marco.celio@unifr.ch](mailto:marco.celio@unifr.ch)

**Supplementary Table 3**

| **Gene Accession** | **Gene symbol** | **Gene description** | **FW Primer** | **RV Primer** | **Size product** |
| --- | --- | --- | --- | --- | --- |
| NM_019626 | Cbln1 | cerebellin-1-precursor | GGTGAAAGTCTACAACAGACAGACCAT | CGGCGAAGGCTGAAATCA | 76bp |
| ENSMUST00000075764 | Drd2 | dopamine 2 receptor | ATCTCTTGCCCACTGCTCTTTGGA | ATAGACCAGCAGGGTGACGATGAA | 131bp |
| NM_173447 | Ephb1 | ephrin b1 receptor tyrosine kinase | CAAGATGTGTTTCCAGACTCTGACA | TGCCGAGCCAGCAATCA | 81bp |
| NM_022378 | Foxb1 | Foxb1 transcription factor | CTACCGGGCTGAGTCCAAG | CGGAGGACTGGTCGAATCTT | 155bp |
| ENSMUST00000058787 | Glra2 | glycine receptor, alpha 2 subunit | GCTAGTGAACATTTTGACAGCC | GGTCTGCGAGGGATGTTTTC | 112bp |
| ENSMUST00000000275 | Glra3 | glycine receptor, alpha 3 subunit | TCTTTCCGTATCATGGCCCA | AGATCGCGCACTGTTTGTTT | 120bp |
| NM_153288 | Npb | neuropeptide B | CGGACCTCTGCGCAATTTAG | CGTCCGCTTTACACTGGAAA | 134bp |
| NM_175678 | Npsr1 | neuropeptide S receptor | GTTCACTGAAGCACTGGTGG | GCACAACAGAGTTTCCCACA | 119bp |
| ENSMUST00000102716 | Glra1 | glycine receptor, alpha 1 subunit | GGGGAGGACTTCTGGGTATG | GTTGTCTCAGCGATGGAACC | 111bp |
| ENSMUST00000100497 | Actb | actin beta | GGCTGTATTCCCCTCCATCG | CCAGTTGGTAACAATGCCATGT | 154bp |

**Legends**

**Supplementary Table 3**:

The qRT-PCR forward (FW) and reverse (RV) primers used in the study are listed in this table, together with the relating gene accessions, gene symbols, gene descriptions and product sizes.

**
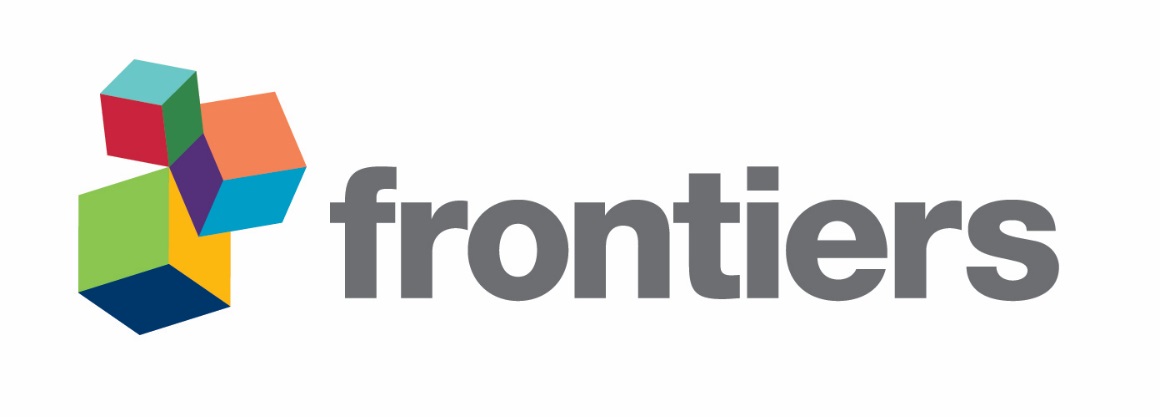
**
